# Circadian Clock, Cell Division, and Cancer: From Molecules to Organism

**DOI:** 10.3390/ijms18040873

**Published:** 2017-04-20

**Authors:** Anton Shostak

**Affiliations:** Circadian Rhythms and Molecular Clocks Group, Heidelberg University Biochemistry Center, 69120 Heidelberg, Germany; anton.shostak@bzh.uni-heidelberg.de; Tel.: +49-6221-54-4342

**Keywords:** the circadian clock, the cell cycle, proliferation, cancer, clock-controlled genes, circadian disruption

## Abstract

As a response to environmental changes driven by the Earth’s axial rotation, most organisms evolved an internal biological timer—the so called circadian clock—which regulates physiology and behavior in a rhythmic fashion. Emerging evidence suggests an intimate interplay between the circadian clock and another fundamental rhythmic process, the cell cycle. However, the precise mechanisms of this connection are not fully understood. Disruption of circadian rhythms has a profound impact on cell division and cancer development and, vice versa, malignant transformation causes disturbances of the circadian clock. Conventional knowledge attributes tumor suppressor properties to the circadian clock. However, this implication might be context-dependent, since, under certain conditions, the clock can also promote tumorigenesis. Therefore, a better understanding of the molecular links regulating the physiological balance between the two cycles will have potential significance for the treatment of cancer and associated disorders.

## 1. Introduction

From the dawn of life on our planet, various organisms have been exposed to periodic variations in different environmental factors, such as sunlight or temperature. The evolutionary advantage of being able to estimate the duration of the day and to predict the occurrence of daily events, has triggered the development of the circadian clock [1]. This idea is supported by laboratory experiments with different strains of the cyanobacterium *Synechococcus elongatus* demonstrating that internal circadian period resonating with exogenous light-dark regimes confers substantial benefits in cellular growth [2].

Circadian clocks are considered to regulate cell division (or reproduction in the case of unicellular organisms) at early evolutionary stages [3]. According to the “escape from light” hypothesis, ancient life forms developed the clock to avoid harmful radiation emitted by the sun [4]. It is quite conceivable that by restricting replication events to the night, the clock would help to avoid the deleterious effects of ultraviolet (UV) light on DNA integrity [5,6,7]. In agreement with this, circadian rhythms in the susceptibility to UV radiation were reported in single-cellular algae (*Chlamydomonas reinhardtii*) as well as in the skin of mammals [7,8,9,10]. Subsequently, as protection to UV-induced DNA damage, different organisms developed specific blue light sensors that are also capable of DNA repair, named photolyases. Interestingly, in many species, a subfamily of enzymatically inactive photolyase homologs, the cryptochromes, are involved in light resetting of the circadian clock [11,12]. Another observation supporting this hypothesis is that UV light can act as a clock entrainment signal, by inducing phase shifts of circadian rhythms in different biological systems [13,14]. Remarkably, a family of tryptophan-based UV receptors (UVR8) identified in plants was demonstrated to mediate synchronization of the *Arabidopsis thaliana* circadian clock to UV light [15,16,17,18].

An alternative hypothesis suggests that the link between the circadian clock and the cell cycle is required to temporally separate DNA replication from oxidative metabolic reactions. Metabolic rhythms in the budding yeast are characterized by respiratory fluctuations with a period of 40 min to 4 h (dependent on strain genotype). They are often considered as a timing mechanism, analogous to the circadian clock [19,20,21]. In order to maintain genome integrity, yeast cells restrict their DNA replication (S) phase exclusively to the reductive stage of the metabolic cycle and allow no DNA biosynthesis during the oxidative stage, when mutagenic reactive oxygen species are produced. In line with this, mutant strains that permit DNA synthesis during the oxidative stage show increased rates of spontaneous point-mutations [22].

Taken together, both scenarios provide plausible explanations for DNA damage acting as the driving force to synchronize the circadian clock and cell cycle regulation [23].

## 2. The Circadian System in Mammals

**Figure 1:**
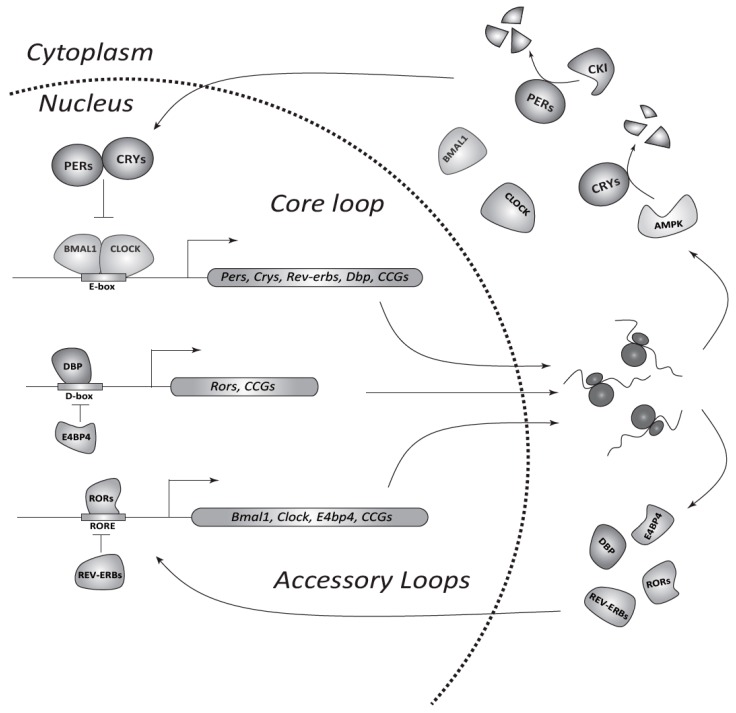
The circadian transcriptional/translational feedback loop (TTFL) machinery in mammals. In the core loop CLOCK/BMAL1 bind *E-boxes* in promoters of target genes (*Per*s, *Cry*s, and clock-controlled genes (CCGs)) and activate transcription. Nuclear export and translation of obtained mRNAs allows gradual accumulation of PERs and CRYs in the cytoplasm. Kinases, such as CKI and 5′ AMP-activated protein kinase (AMPK), adjust the period of the clock by phosphorylation and subsequent degradation of PER and CRY proteins, respectively. PERs and CRYs form complexes, which enter the nucleus and inhibit CLOCK/BMAL1-mediated transcription. Consequent degradation of PERs and CRYs restarts a new cycle of transcription. Accessory loops contain additional pairs of antagonizing transcription factors such as REV-ERBs (α/β) and RORs (α/β/γ), or DBP (*D-box*-binding protein) and E4BP4 (E4 promoter-binding protein 4). The former regulates *Clock* and *Bmal1* genes through *ROR*-elements (*RORE*), whereas the latter controls the expression of other CCGs via *D-boxes* at a second hierarchical level.

The circadian timing system in mammals is organized in a hierarchical manner, with a central oscillator in the brain and peripheral oscillators in virtually all cells of the body. In mammals, the central clock is located in neural networks of the hypothalamic suprachiasmatic nuclei (SCN) which receive photic information from the retina and synchronize peripheral clocks with external light/dark cycles via neural and humoral pathways [24]. On the cellular level, the molecular clockwork in plants, fungi, and metazoans is based on transcriptional/translational feedback loops (TTFLs) compiled of so-called clock genes [25,26]. In the center of mammalian TTFLs, there are two *E-box* specific transcription factors, CLOCK (Circadian locomotor output cycles kaput, which can be replaced by NPAS2 (Neuronal PAS domain protein 2)) and BMAL1 (Brain and muscle Arnt-like protein-1), which, at the beginning of the day, form heterodimers, and bind and activate transcription of target genes (Figure 1). Their targets include a small group of genes encoding transcriptional repressors, the *Period* (*Per1*/*2*/*3*) and *Cryptochrome* genes (*Cry1*/*2*). Freshly translated PER and CRY proteins form heterocomplexes, which gradually accumulate in the cytoplasm and interact with casein kinase I (CkIδ and CkIε) and 5′ AMP-activated protein kinase (AMPK). Subsequently, phosphorylated CRYs and PERs are degraded via the proteasome pathway to ensure a required temporal delay of the negative arm of the TTFL. Later in the evening, PER/CRY complexes enter the nucleus and inactivate CLOCK/BMAL1 dimers, thus inhibiting the transcription of their own genes and closing the loop. In addition to *Per*s and *Cry*s, CLOCK/BMAL1 drive rhythmic expression of many clock-controlled genes (CCGs) directly or via transcription factors of secondary loops, such as REV-ERBs (reverse strand of ERBA)/RORs (Retinoic acid-receptor-related orphan receptor) and others [27]. This network of TTFLs allows for the expansion of the number of CCGs to reach up to 10–15% of the whole transcriptome in a given tissue [28]. Moreover, recent phosphoproteomic analysis revealed that timing information could be further conducted to various signaling pathways by means of circadian changes in phosphorylation [29].

## 3. The Cell Cycle in Mammals

**Figure 2:**
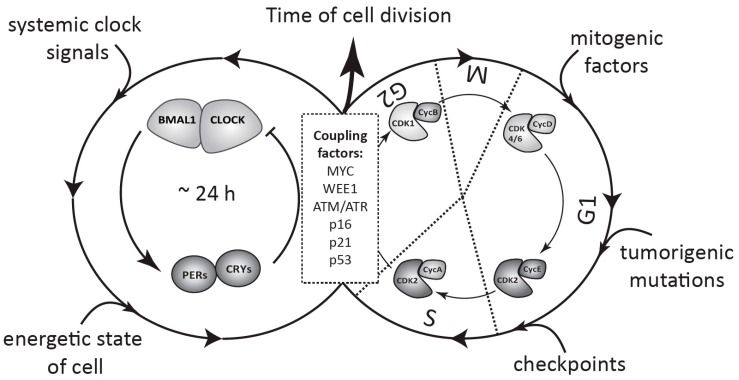
Schematic view of coupling between the circadian clock and the cell cycle in healthy and tumor cells. The circadian oscillator based on TTFLs and the cell cycle, consisting of several phases regulated by CDK/cyclin complexes, coexist in a single cell. The clock is entrained by systemic signals from the body and by the internal energetic state of the cell, whereas cell cycle progression depends on a combination of other factors, such as mitogenic stimulation, tumorigenic mutations, and DNA damage checkpoints. Interaction of both oscillators, as defined by specific coupling factors, determines the circadian timing of cell division (for details see Section 3 and Section 4).

The cell cycle is a continuous process of cell growth and DNA duplication, followed by cell division (mitosis). It consists of several cell cycle phases. The transition from one phase to another is controlled by a set of conserved serine-threonine cyclin dependent kinases (CDKs), whose activity is regulated by special adaptor proteins—cyclins—expressed in a temporal manner (Figure 2). According to the classical model, entry into G1 phase is controlled by a complex of CDK4/6 with D cyclins (CycD), which phosphorylates retinoblastoma protein (Rb) and releases the E2F (E2 factor) transcription factor. E2F, in turn, activates expression of the cyclins E and A. The transition from G1 to S phase is controlled by CDK2 in complex with cyclin E, which is later replaced by cyclin A, to initiate S phase. The S to G2 transition and M phase are controlled by CDK1 in complex with cyclins A and B, respectively. The activity of each of the CDK complexes can be restrained at each phase by a set of specific inhibitors such as p15, p21, p27, or WEE1 [30].

It is worth mentioning, however, that according to results of knockout studies in mice, the interphase CDKs (CDK2/4/6) are directly required not for the cell cycle in general, but for the development of specific cell types [31,32,33,34,35]. Only the deletion of mitotic CDK1 results in cell cycle arrest and early embryonic lethality. Similar to yeast, mammalian CDK1 is able to bind all types of cyclins and maintain proliferation in embryos until mid-gestation [35]. These results challenged the classical view and led to the development of the “essential” cell cycle model, in which CDK1 plays a central role [30].

Correct cell cycle progression is dependent on several checkpoints, which are activated in response to DNA damage and induce cell cycle arrest to avoid transmission of altered genomes to daughter cells. Double strand breaks during G1 phase induce activity of ataxia-telangiectasia mutated (ATM), which phosphorylates checkpoint kinase 2 (CHK2) and activates p53, preventing cells from proceeding into DNA replication. DNA damage during the S/G2 phases results in activation of ATR (ATM- and RAD3-related) kinase, which signals through CHK1 and p53, leading to cell cycle arrest [36].

## 4. Molecular Links between the Circadian Clock and the Cell Cycle

In their seminal work, Matsuo and colleagues studied circadian aspects of liver regeneration after partial hepatectomy. They demonstrated that rhythmic expression of the WEE1 kinase (which inhibits the G2/M transition by phosphorylation of CDK1) is transcriptionally governed by CLOCK/BMAL1, leading to delayed mitosis entry after injury in circadian mutant mice (*Cry1,2^−/−^*) [37]. Similarly, the p53 tumor suppressor pathway was found to be under direct transcriptional control through BMAL1, which is consistent with an antiproliferative role of BMAL1 in pancreatic cancer [38]. The circadian output effector NONO was shown to associate with PERIOD proteins to directly activate the cyclic expression of p16^INK4A^ (which inhibits the G1/S transition) and to regulate cell cycle progression in a circadian fashion [39]. Another CDK inhibitor, p21^Cip1^, was shown to be rhythmically regulated via the REV-ERB/ROR loop, through conserved *RORE* motifs in its promoter [40]. Remarkably, DNA damage was revealed to affect the turnover of both cryptochromes in an opposite manner by increasing stability of CRY1 and concomitantly destabilizing CRY2. Since both CRYs appear to have a non-redundant function in this process, a precise balance between them is required to shape the proper transcriptional response to genotoxic stress [41].

Post-translational modifications further contribute to the coupling between the two oscillators. In the unicellular red alga *Cyanidioschyzon merolae*, this link was ultimately narrowed down to time-dependent phosphorylation of the transcription factor E2F, which regulates the G1/S transition. Remarkably, mutation of E2F phosphorylation sites results in uncoupling of cell divisions from the circadian clock [42]. A recent report unexpectedly revealed a unique function of CRY2 as a key factor of MYC (avian myelocytomatosis viral oncogene homolog) turnover. In cooperation with FBXL3 (F-box and leucine rich repeat protein 3), CRY2 binds to MYC phosphorylated at threonine 58 and targets it for degradation, thus restricting proliferation in cancer cells [43]. Another member of the clock’s negative feedback loop, PER2, is involved in the regulation of p53 stability. PER2 binding hinders Mdm2-mediated ubiquitination of p53 and facilitates its nuclear import, while rendering it transcriptionally inactive. This generates a precondition, when p53 levels in the nucleus are instantly accessible for the immediate reaction to genotoxic stress [44,45,46]. PER1, in turn, controls phosphorylation of CHK2 via direct interaction with ATM and, thus, enhances cell cycle arrest and apoptosis upon DNA damage [47]. Although the role of mammalian timeless (TIM) in the clock mechanism remains unclear, its function in both limbs of DNA damage responses seems to be crucial [48,49,50]. On the one hand, TIM facilitates phosphorylation of CHK1 by ATR in response to UV irradiation or hydroxyurea treatment [51]. On the other hand, TIM is responsible for the activation of CHK2 by ATM in response to doxorubicin-induced DNA double strand breaks [52]. Despite substantial progress, the identification of precise molecular mechanisms of coupling in particular tissues or tumors still remains a challenging task, since cell cycle regulation possesses a considerable reserve of plasticity, due to the redundancy of its individual components.

## 5. Coupling between the Circadian Clock and the Cell Cycle

Circadian rhythms in cell division were documented in various biological systems, such as cyanobacteria and unicellular eukaryotes, suggesting a link between two oscillators [7]. Based on these observations, it was proposed that the circadian oscillator could act as an additional checkpoint, allowing (“gating”) cell divisions only during certain time windows [53]. Indeed, in cyanobacteria, cell proliferation shows clear gating by the circadian clock, although the period of cell division cycles is much shorter than one day (around 10 h) [54,55]. Studies in humans and mice also report circadian variations in DNA replication and rhythmic expression of cell cycle components in different tissues in vivo [56,57,58,59]. Development of fluorescent circadian reporters allowed for investigation of circadian rhythms in isolated cells devoid of systemic cues. In NIH 3T3 cells, the incidence of cell division events relative to the circadian cycle is highly non-random and exhibits a trimodal frequency distribution, suggesting that cellular clocks predetermine the timing of mitosis [60]. Subsequent mathematical analyses confirmed the intimate link between both oscillators in NIH 3T3 cells [61,62]. Interestingly, both experimental data and stochastic modelling defined the nature of this interaction as a 1:1 phase locking (i.e., oscillations with a common frequency) rather than a gating, as suggested earlier [53,61,62]. Another remarkable finding was reported by a recent study using complex tissue culture techniques such as 3D intestinal organoids. The authors revealed that intercellular coupling helps stem cells to synchronize their cell divisions with local circadian pacemakers residing in secretory Paneth cells. Rhythmic secretion of Wnt by Paneth cells entrains the cell cycle of adjacent stem cells and progenitor cells bearing weak circadian oscillators [63].

Similar to what is observed in cellular circadian clocks, genetically identical cells tend to show high variabilities in cell cycle durations [64]. Are the factors responsible for this variability stochastic and set randomly, independent of initial conditions, or deterministic and rely on certain inherited components? Recently, Sandler et al. addressed this issue in an elegant experimental approach comparing cell cycle durations among different lineages of cells. They found high correlations in pairs of sister and cousin cells, and no correlation between mother and daughter cells [65]. This property, termed the “cousin-mother inequality”, suggests deterministic inheritance within the system. A “kicked cell cycle” model, built on this experimental data, assumes the existence of a certain independent oscillator, whose phase after each cell division would determine the duration of the next cell cycle. It is still unclear whether this underlying oscillator is the circadian clock, but some hints, such as inheritance of the circadian phase by the daughter cells upon division, point in this direction [60,65,66].

In contrast, certain cell types show a marked absence of coupling between the clock and the cell cycle. In rat1 fibroblasts, luciferase activity of the cell cycle reporter (*CCNB1-dGluc*) is rhythmic but not temperature compensated, and does not correlate with oscillations of the circadian *Bmal1-dGluc* reporter [67]. Similar phenomena are observed in Lewis lung carcinoma cells [68]. Taken together, both oscillators show robust coupling in vivo and in vitro; however, under certain conditions, immortalized or cancer cell lines uncouple their cell division from the circadian control.

## 6. Physiological Significance of the Clock-Cell Cycle Coupling

In the adult body, division of many stem cells is controlled by the circadian clock. Diurnal mitotic rhythms in UV exposed tissues, such as skin, were among the first to be reported [69,70]. Later studies provided compelling evidence that the circadian clock indeed plays a crucial role in the physiology of epidermal stem cells. For instance, healthy skin homeostasis requires a balance between pools of dormant and active skin stem cells, which is in turn determined by the local clock. Disruption of clock genes in these cells results in premature epidermal ageing and predisposes to cancerogenesis [71]. The circadian clock found in human keratinocytes temporally regulates expression of a large number of genes involved in proliferation, sensitivity to signaling pathways, and DNA damage responses [72,73]. Moreover, rhythmic clock gene expression was reported in another constantly remodeling human organ, the hair follicle. Disruption of the clock components *Per1*, *Bmal1*, or *Clock* with RNAi significantly prolongs the anagenic phase of intensive epithelial proliferation, suggesting that the clock is required for a normal progression of the hair cycle [74]. Multiple studies provide evidence that the molecular clockwork is important for normal stem cell function in other organs also, such as brain, blood, and intestine [75]. The activation of quiescent neuronal progenitor cells shows time-of-day dependent fluctuations, which require intact clock genes. Genetic ablation of circadian rhythms disrupts proper adult hippocampal neurogenesis, leading to impaired cognitive functions such as learning and memory [76]. Clock-controlled release and accumulation of hematopoietic stem cells and inflammatory monocytes in the circulating blood is important for the regeneration of the stem cell niche in bone marrow and the modulation of inflammatory reactions, respectively [77,78]. Divisions of intestinal stem cells, necessary for efficient renewal of the crypt epithelium after lining, are stimulated by circadian Wnt secretion from Paneth cells and require an intact circadian clock (see above) [63].

## 7. The Circadian Clock as a Tumor Suppressor

In the pathological state, a loss of cell cycle regulation leads to uncontrolled cell division and, ultimately, the development of cancer. Whether and to what extent circadian clocks are involved in this process remains a high-priority question. Nowadays, the prevailing hypothesis states that the circadian clock is an important tumor suppressor, and that disrupted circadian rhythms promote tumor development [79]. Population studies conducted on different cohorts of subjects associate shift work and insufficient sleep with an elevated risk of cancer development [80,81,82,83,84,85]. Results from modeling circadian disruption in rodents by SCN lesion or by aberrant light schedules such as shift work and chronic jet lag under controlled laboratory conditions support these observations. Cohorts of tumor-bearing mice, subjected to such treatments, show accelerated tumor growth and increased expression of genes involved in tumorigenesis such as *Myc* [86,87]. Additionally, tumor-prone mice, expressing a mutated allele of p53 in mammary glands, exhibit higher rates of spontaneous tumors, when exposed to weekly alternating light cycles, suggesting that internal desynchronization and sleep disturbances contribute to de novo cancerogenesis [88].

Numerous studies reported that individual molecular components of the circadian clock, such as BMAL1 [38,89,90,91,92,93], PER2 [94,95,96], or PER1 [47] suppress proliferation or increase the sensitivity to anti-cancer drugs in different cancer cell lines. Moreover, enhancing the clock function in tumor cells by means of circadian synchronization (i.e., dexamethasone treatment) impinges on the cell cycle and reduces cellular growth [97]. In line with this, genetic variants of various clock genes were associated with certain types of cancer in humans (reviewed in [98,99]).

If an intact circadian clock indeed acts as a tumor suppressor, then mutations of clock genes in mice should predispose to cancerogenesis and lead to higher tumor frequencies. Initial work performed by Fu and colleagues supports this hypothesis, revealing that *Per2^m^*^/*m*^ (and also *Per2^−^*^/*−*^) mice are sensitive to DNA damage and subsequent tumor development induced by γ-radiation [100]. Analogously, another group found that downregulation of PER2 increases proliferation of colon cancer cell lines, and *Per2^m^*^/*m*^ mice are prone to formation of precancerous polyps in colon. This phenotype is further aggravated upon mutation of *Per2* in mice inclined to develop intestinal tumors (*Apc^Min/+^*) [101]. Furthermore, long-term observations of animals with single or double deletions of clock genes such as *Per1,2^−^*^/*−*^ and *Cry1,2^−^*^/*−*^ or mice lacking a single copy of *Bmal1*, revealed them to be cancer-prone. All three genotypes developed significantly more spontaneous and radiation-induced tumors than wild type animals already under normal 12:12 light:dark conditions, and this phenotype was further augmented when animals were phase-shifted [102]. The tumor-suppressive potential of intact circadian rhythms was demonstrated using mice models of induced lung cancer (*K-ras^LSL−G12D/+^*; *p53^flox/flox^*). Either subjected to chronic jetlag or bearing mutated alleles of *Per2* or *Bmal1*, these animals showed increased tumorigenesis and lower survival rates, which correlated with higher proliferation rates and MYC expression levels in their tumors [103]. A recent study underlined the role of circadian dysfunction in the development of non-alcoholic fatty liver disease and liver cancer in obese people [104]. Wild type animals subjected to chronic jet lag shifted their liver metabolism towards lipid synthesis and storage, leading to the development of steatohepatitis, fibrosis, and, ultimately, hepatocellular carcinomas. Interestingly, double mutation of cryptochromes (*Cry1,2^−^*^/*−*^), Periods (*Per1,2^−^*^/*−*^), or *Bmal1* in the liver (*Alb^cre^*; *Bmal1^fl^*^/*fl*^) accelerates progression of these symptoms and increases tumor incidence [104].

However, there were also studies which report contradicting results, and assert that clock gene mutant mice are not tumor prone. In contrast to previous reports, the rate of spontaneous tumors in untreated and γ-irradiated *Per1^−^*^/*−*^ and *Per2^−^*^/*−*^ mice is comparable to that of wild type animals [105]. Moreover, arrhythmic *Bmal1*^−/−^ and *Clock^∆19^* mutants also do not show higher tumor frequencies, although they develop symptoms of accelerating aging under normal conditions (*Bmal1^−^*^/*−*^), or when subjected to γ-radiation (*Clock^∆19^*) [106,107]. A similar discrepancy was reported for *Cry1/2^−^*^/*−*^ knockout animals, which are equally susceptible to spontaneous or radiation-induced cancers as wild types [108]. Strikingly, deletion of both cryptochromes was described to have a cancer protective effect, as it significantly reduces mortality and tumor incidence in mice with *p53^−/−^* background [109]. Despite the discrepancies between different studies, it is conceivable that the observed cancer prone phenotypes of certain clock gene mutants might stem not from the disrupted circadian rhythms per se, but rather from “clock-unrelated” (pleiotropic) functions of these genes [79].

## 8. Does the Circadian Clock Support Tumorigenesis?

The prevailing view regarding the antitumor activity of the circadian clock was disputed by reports that some clock genes support proliferation in normal and cancer cells. Indeed, the expression of many cell cycle genes is deregulated in *Clock^∆19^* mutant mice and, as a consequence, *Clock^∆19^* mouse embryonic fibroblasts (MEFs) exhibit significantly lower proliferation rates than those from wild types [110]. Similarly, *Bmal1* deficiency in primary hepatocytes results in reduced rates of cell division and, vice versa, BMAL1 overexpression stimulates cell growth in NIH 3T3 cells [40,111]. This pro-proliferative effect of certain clock genes was not specific to untransformed cells, since very similar properties were also attributed to cancer cell lines. For instance, human colorectal cancers often show higher expression of *Clock* or *Bmal1* genes compared to healthy tissue [112,113,114]. In agreement with this, overexpression of CLOCK increases proliferation of colorectal carcinoma cells in vitro and in vivo [115]. Another study reports elevated levels of CLOCK in ERα-positive breast tumor samples. Furthermore, upregulation of *Clock* transcription by estrogen receptor (ER) was necessary to maintain high proliferation in tumor cells [116]. BMAL1 was found to be upregulated in certain types of pleural mesothelioma, and subsequent experiments revealed reduced cell growth and induced apoptosis upon *Bmal1* knockdown in tumorigenic cells, but not in cells derived from healthy tissue [117,118]. Finally, both *Clock* and *Bmal1* were identified as survival factors for leukemia stem cells, since their genetic (RNAi, CRISPR) or chemical (REV-ERBs agonist SR9011) disruption induces differentiation and growth arrest. Surprisingly, healthy cells appear to be resilient to genetic ablation of the circadian clock, as *Bmal1* disruption does not produce any gross hematopoietic deficits, thus revealing *Bmal1* as an attractive anti-leukemia target [119].

Taken together, these findings indicate that under certain circumstances, clock genes may foster cancer development and, therefore, their role as tumor suppressors must be re-evaluated. Although the reason remains unknown, it is tempting to speculate that unique epigenetic signatures of various cancer cell types are likely to define distinct subsets of CCGs, modulating influence of the circadian clock on proliferation, apoptosis, and cell cycle progression.

## 9. Cancer Affects Circadian Rhythms in Cells and in the Body

Multiple reports suggest that malignant transformation is associated with suppression of circadian rhythms in tumors [79]. Indeed, many oncogenic pathways have established connections to the circadian clock and can impinge on its function through transcriptional and post-translational mechanisms [120]. Increased activity of the Ras pathway observed in human malignancies weakens circadian oscillations in cells [121]. Overexpression of MYC oncogenes found in many cancers was shown to silence the circadian clock in different types of tumors [93,122,123,124]. The cancer/testis antigen PASD1 (PAS domain containing 1) induced in certain variants of tumors directly interacts with CLOCK/BMAL1 dimers and inhibits their transactivation activity, thereby interfering with the circadian clock [125]. Depending on the mechanism of oncogenic transformation, cancer cells can silence their clock in order to escape rhythmic regulation of metabolism imposed by the circadian system and thereby accelerate cell growth. Alternatively, to increase proliferation, a tumor may uncouple its cell cycle from circadian regulation, as mentioned above [68].

Surprisingly, the process of malignant transformation can affect circadian rhythms in distal organs and introduce imbalance in metabolic homeostasis of the body. Arrhythmic liver metastases of colorectal cancer phase-shift the expression of clock genes in healthy liver tissue. Similar phase shifts, most likely caused by some humoral factors, were also observed in more distal organs such as kidney [126]. Moreover, a recent study reports that mice bearing adenocarcinomas in lung show an altered hepatic circadian metabolism. Although the expression of core clock genes remains unchanged, lung tumors massively affect oscillations of liver metabolic genes and molecules [127]. Furthermore, systemic effects, such as reduced serum insulin and increased blood glucose, hint at a pernicious influence on other peripheral organs such as pancreas. As was suggested, the detrimental impact of tumors is mediated by secretion of proinflammatory cytokines and metabolites such as lactate. In this fashion, tumors may shape the physiology of the host in accordance with their energetic requirements [127].

Another interesting aspect is the systemic impact of hormone-producing tumors on the circadian rhythms in the body. For instance, patients with Cushing syndrome often bear cortisol- or ACTH (Adrenocorticotropic hormone)-secreting tumors associated with sleep disorders and disturbed circadian rhythms [128]. In line with this, patients with pheochromocytoma (adrenaline-producing tumors) exhibit reduced circadian variations in blood pressure [129,130]. Future studies should address whether and how ectopic tumor secretion of circadianly active substances may cause desynchronization of peripheral oscillators and lead to secondary metabolic disturbances and sleep disorders.

## 10. Conclusions

The circadian clock and the cell cycle are two essential rhythmic programs that regulate major aspects of mammalian physiology. Cumulative evidence suggests multiple means by which these oscillators can affect each other in healthy and pathological states. The circadian clock is known to regulate expression of cell cycle components on cellular or intercellular levels and, thereby, gate the cell cycle. Circadian disruption, in turn, results in deregulated cell division and cancerogenesis. However, the exact contribution of already established links versus as-yet-unknown mechanisms remains obscure. On the other hand, malignant transformation and tumor development interfere with molecular clock function and introduce a systemic imbalance in circadian rhythms. Shift work, jet lag, and sleep disorders are inevitable attributes of modern human society, all of which are associated with the development of cancer—the leading cause of mortality worldwide. Therefore, a better notion of the machinery which interconnects these pathological conditions will help to devise new therapeutic strategies for the treatment and prevention of cancer.
